# Embolization of spontaneous retroperitoneal bleeding fed by aneurysmal arc of Bühler: Case report and technical considerations

**DOI:** 10.1016/j.jvscit.2026.102243

**Published:** 2026-03-30

**Authors:** Salvatore Silipigni, Agatino Cacciola, Antonella Cinquegrani, Marcello Dattola, Alberto Stagno, Antonio Bottari

**Affiliations:** Interventional Radiology Unit - Department of Biomedical and Dental Sciences and Morphological and Functional Imaging, University of Messina, A.O.U. Policlinico ‘G. Martino’, Messina, Italy

**Keywords:** Arc of Bühler, Aneurysm, Embolization

## Abstract

The arc of Bühler (AOB) is a rare embryonic anastomosis between the celiac artery and the superior mesenteric artery, which may become a crucial collateral pathway in cases of celiac axis stenosis. We report a challenging case of a 70-year-old woman presenting with hemorrhagic shock owing to a bleeding branch of an aneurysmal and highly tortuous AOB, with median arcuate ligament compression of the celiac trunk. Endovascular treatment was performed successfully by selective embolization of the bleeding branch with the sacrifice of the AOB using a Penumbra Occlusion Device and packing coils, preserving celiac-mesenteric circulation via the pancreaticoduodenal arcade. This case highlights the importance of recognizing vascular variants and adapting embolization strategies to complex anatomy in emergency settings, reinforcing the pivotal role of interventional radiology in managing visceral aneurysms and acute abdominal hemorrhage.

The arc of Bühler (AOB) is a rare persistent embryonic anastomosis between the celiac artery and the superior mesenteric artery (SMA). Its presence may serve as an important collateral pathway in cases of pancreaticoduodenal arcade failure; if unrecognized, however, it poses a significant risk for major hemorrhagic complications during duodenopancreatic surgery.^1^^,^^2^

True aneurysms of the AOB, characterized by preserved arterial wall layers, are even more uncommon and are typically associated with celiac axis stenosis, either owing to atherosclerosis or median arcuate ligament compression.^3^ This results in collateral hyperflow and progressive arterial dilatation. Conversely, pseudoaneurysms usually arise from iatrogenic injury, postoperative complications, or pancreatitis-induced arterial wall erosion.^4^

Clinical presentation can range from incidental findings to acute hemorrhage with hemodynamic instability. Current guidelines recommend treatment for true visceral artery aneurysms ≥15 mm and urgent intervention for all pseudoaneurysms.^5^^,^^6^

The contemporary management of visceral artery aneurysms, including aneurysmal AOB, involves either surgical repair or endovascular embolization. Endovascular approaches such as coil embolization, liquid embolic agents, or covered stent placement are increasingly preferred owing to high technical success rates and reduced invasiveness. These methods aim to exclude the aneurysm while preserving the celiac-mesenteric circulation. However, treatment may be complicated by anatomical tortuosity, which can hinder catheterization or increase the risk of coil migration.^4^^,^^7^

## Case report

**Fig 1:**
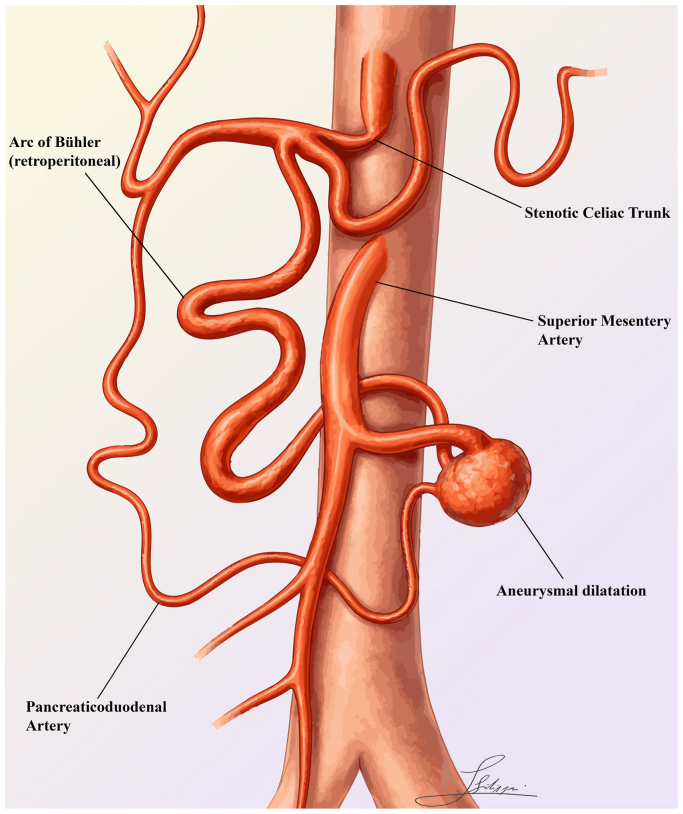
Simplified anatomical illustration of the anatomical variant with dual connection of mesenteric artery and celiac trunk with pathologic pancreaticoduodenal artery and patency of the arc of Bühler (AOB).

A 70-year-old woman presented to the emergency department of an outside hospital with acute abdominal pain, hypotension, and tachycardia. Computed tomography angiography revealed active retroperitoneal and intraperitoneal contrast extravasation with hemoperitoneum and severe stenosis of the celiac axis owing to median arcuate ligament compression, clinically silent owing to compensation through the pancreaticoduodenal arcade and a patent AOB. The latter was ectatic and highly tortuous, and the former presented an irregular diameter and a true aneurysmal dilatation at its inferior origin from the SMA (Fig 1).

The patient was subsequently transferred to our department. Selective angiography of the celiac axis, performed through right 5F femoral access, confirmed severe stenosis. Despite several attempts the vessel was crossable only with an 0.018-inch guidewire, but not with standard angiographic microcatheters. Fearing dissection of the celiac artery with further maneuvers and given that stenting of the celiac axis was not considerable owing to extrinsic anatomical compression, the mesenteric route was preferred.^6^

**Fig 2:**
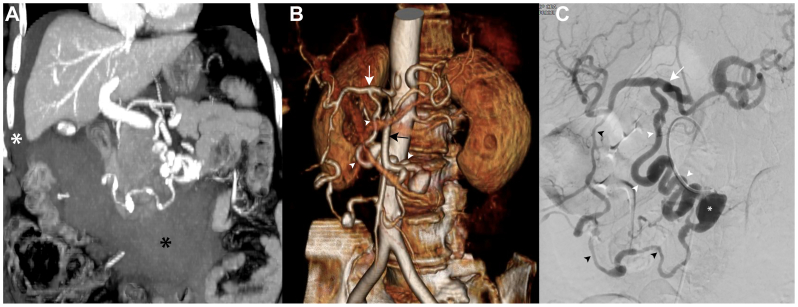
**(A)** Preoperative computed tomography angiography (2.5 mm thick; maximum intensity projection oblique coronal reconstruction) showing a massive retroperitoneal hemorrhage (*black asterisk*) and perihepatic hemoperitoneum (*white asterisk*). **(B)** Volume reconstruction depicting the ectatic and tortuous arc of Bühler (AOB) connecting the superior mesenteric artery (SMA) (*black arrow*) and the celiac trunk (*white arrow*). The AOB (*white arrowheads*) runs behind SMA owing to his retropancreatic position. **(C)** Digital subtraction angiography performed from SMA showing the double connection between the celiac axis (*white arrow*) and SMA (point of injection) by the tortuous AOB (*white arrowheads*) and the pancreaticoduodenal arcade (*black arrowheads*) characterized by irregular diameter. The angiogram shows a 22-mm true aneurysm (*white asterisk*) at the inferior origin of the pancreaticoduodenal arcade.

**Fig 3:**
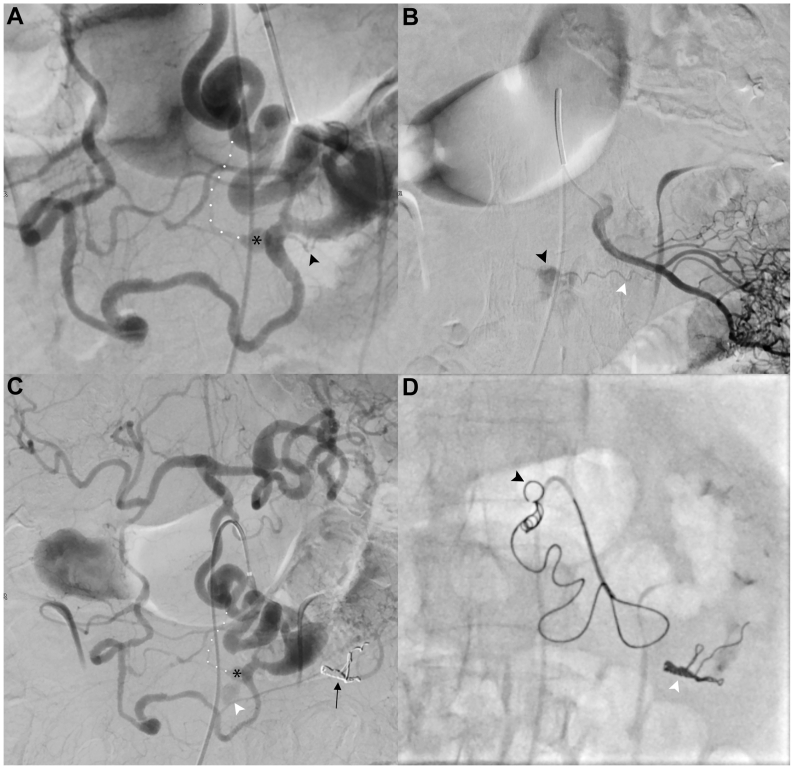
**(A)** Selective digital subtraction angiography (DSA) of the arc of Bühler (AOB) and pancreaticoduodenal artery (injection from the superior mesenteric artery [SMA]). The angiogram shows the bleeding retroperitoneal pseudoaneurysm (*black asterisk*) supplied by a thin feeder from a jejunal branch of SMA (*black arrowhead*) and a thin retroperitoneal feeder originating from the mid-portion of the AOB (*white dotted line*). **(B)** Selective DSA of the jejunal branch confirmed the subtle supply (*white arrowhead*) to the pseudoaneurysm (*black arrowhead*) surrounded by suffusion of contrast medium owing to active bleeding. **(C)** DSA after embolization of the SMA branch showing the subtracted coil cast (*black arrow*) and the persistence of the pseudoaneurysm (*black asterisk*) with hemorrhagic suffusion (*white arrowhead*) supplied by a thin feeder arising from the mid-portion of the AOB (*white dotted line*). **(D)** Fluoroscopic frame during the backdoor phase of AOB embolization. The distal tip of the 5-mm Penumbra Occlusion Device (*black arrowhead*) anchored to the AOB wall allowed controlled and safe deployment of the soft part of the coil. On the left lower corner of the figure is visible the coil cast previously used to embolize the SMA branch (*white arrowhead*).

Selective SMA angiography was performed using a 5F Cobra C2 catheter (Cordis Endovascular) (Fig 2). The angiographic study confirmed a tortuous pancreaticoduodenal arcade with ectasia and aneurysmal dilatation at the origin from the SMA (maximum diameter, 22 mm). The retroperitoneal bleeding took origin from a pseudoaneurysm fed by a subtle feeder branching from the midportion of the AOB, with an additional subtle feeder from a jejunal branch of the SMA (Fig 3).

Selective catheterization of the jejunal vessels using a 2.7F microcatheter (Progreat, Terumo) confirmed the feeder branch. Superselective embolization of this vessel was performed with two microcoils (Ruby coil soft; Penumbra), achieving successful occlusion without ischemic consequences on the bowel loop, thanks to collateral compensation via the marginal artery.

A subsequent angiogram revealed the origin of the second subtle feeder, arising from the midportion of the AOB. Precise identification and selective catheterization of this branch was precluded both from above, because it was impossible to cross the celiac stenosis, and access via the SMA was complicated by marked tortuosity. As a result, embolization was performed by sacrificing the AOB with a sandwich technique: the upper part of the AOB (with a mean diameter of 5 mm) was navigated distal to the origin of the target vessel and occluded using a 5-mm Penumbra Occlusion Device, followed by the deployment of soft liquid metal coils (Packing Coil, Penumbra), ensuring stable distal and proximal occlusion without coil migration.

**Fig 4:**
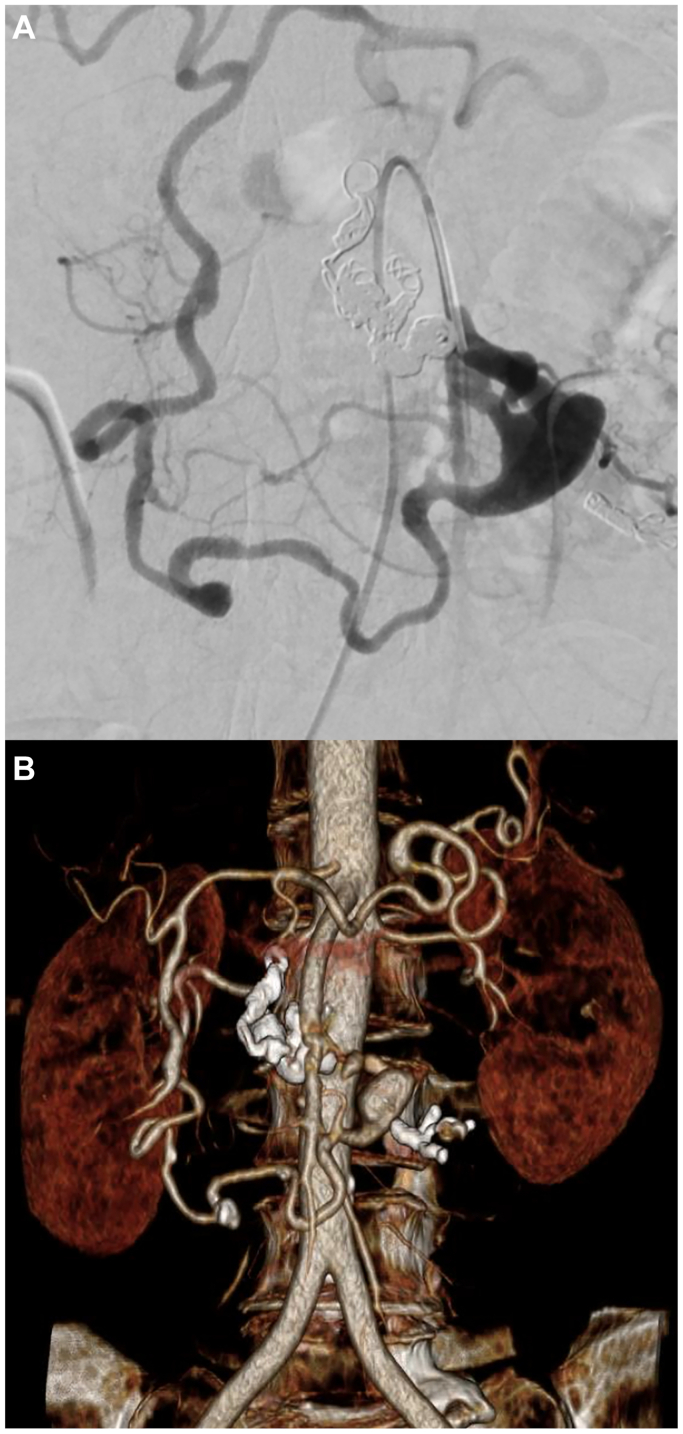
**(A)** Postembolization digital subtraction angiography shows the subtracted cast of coils after packing of the arc of Bühler (AOB), exclusion of the pseudoaneurysm and preserved patency of the celiac-mesenteric collateral circulation. **(B)** Volume reconstruction of the 7-day follow-up computed tomography angiography confirming the postembolization outcome, with maintained patency of the celiac-mesenteric circulation.

Collateral celiac-mesenteric circulation was preserved through the pancreaticoduodenal arcade, confirmed on both the final angiogram and follow-up computed tomography angiography performed 7 days later (Fig 4).

Embolization of the 22-mm aneurysm at the origin of the pancreaticoduodenal artery was postposed to a second session, to be planned after the recovery from the hemorrhagic shock.

The patient agreed to having this case and associated image published. All procedures performed in studies involving human participants were in accordance with the ethical standards of the institutional and/or national research committee and with the 1964 Helsinki declaration and its later amendments or comparable ethical standards.

## Discussion

Most reported hemorrhagic complications arising from an aneurysmal AOB involve chronic celiac axis occlusion leading to collateral hyperflow. The retroperitoneal location of the AOB increases the technical complexity and risk of open surgical repair, particularly in emergency settings with major hemorrhage and high associated mortality.^8^^,^^9^

Initial attempts at endovascular management of AOB aneurysms were limited by the lack of technical options available to pioneers of embolotherapy, as illustrated by Kugai et al^4^ However, endovascular techniques are now considered the first-line approach owing to their lower complication rates and minimally invasive nature.^10^

Device selection for embolization is influenced by several factors, including aneurysm type (true vs pseudoaneurysm), vascular anatomy, and flow dynamics.^4^ Hemodynamic instability in acute bleeding scenarios also significantly impacts treatment decisions. Mechanical embolic agents are generally favored for target occlusion as they are user friendly, controllable, and repositionable; in contrast, liquid embolic agents are perfect for peripheral embolization where distal penetration is allowed, but their use requires experience owing to the risk of nontarget embolization, especially in large diameter vessels with high flow regimens.

Coiling, with or without balloon assistance remains the most commonly used technique for true saccular aneurysms.^7^^,^^11^ Quaretti et al^10^ reported successful embolization of an AOB aneurysm using covered stent-assisted coiling; however, the use of covered stents in emergency settings is generally weighed against the bleeding risk associated with dual antiplatelet therapy.

We report a particularly challenging case of massive retroperitoneal hemorrhage originating from a small branch of an aneurysmal, highly tortuous AOB. The significant tortuosity and the 22-mm aneurysmal dilatation at the origin of the AOB made selective catheterization of the thin branch unfeasible. The decision to sacrifice the AOB was driven by these anatomical constraints.

Safe embolization of the affected arterial segment was achieved by navigating beyond the origin of the bleeding branch using highly trackable materials for sandwich embolization (also known as backdoor/frontdoor embolization), a technique involving the embolization of both the distal and proximal afferences to a target vessel, to prevent collateral reperfusion.^12^ This was accomplished using the Penumbra Occlusion Device, a hybrid coil designed specifically for high-flow vessel occlusion. Its three-dimensional shape allows optimal wall apposition and secure occlusion.

Alternative occlusion devices, such as Amplatzer Vascular Plugs or Micro Vascular Plugs, were unsuitable because vessel tortuosity would have limited distal navigation. Cohesive liquid embolic agents (eg, ethylene vinyl alcohol) could have been considered, but the need for preparation (shaking), the patient's unstable condition, and inability to contain penetration in the high flow regimen of the AOB precluded their use. The decision to sacrifice the AOB was justified by the preserved celiac-mesenteric collateral circulation through the pancreaticoduodenal arcade.

A potential strategy for the embolization of the true aneurysmal segment of the pancreaticoduodenal artery to preserve this critical collateral pathway could involve the use of a flow-diverting stent.

## Conclusions

This case and literature review highlight the clinical significance of chronic celiac trunk occlusion and the complex vascular anomalies that may arise as compensatory collateral pathways. These present substantial challenges for interventional radiologists, particularly in emergency hemorrhagic settings. This case underscores the importance of preprocedural anatomical assessment and individualized treatment planning. With the expanding range of endovascular tools available, interventional radiology continues to play a leading role in the management of visceral artery aneurysms and hemorrhages. Future innovations may further enhance outcomes in this evolving field.

## Funding

None.

## Disclosures

None.
